# Androgen deprivation therapy and fracture risk in Chinese patients with prostate carcinoma

**DOI:** 10.1371/journal.pone.0171495

**Published:** 2017-02-03

**Authors:** Chi-Ho Lee, Gang Huang, Pak-Hei Chan, Jojo Hai, Chun-Yip Yeung, Carol Ho-Yi Fong, Yu-Cho Woo, Kwan Lun Ho, Ming-Kwong Yiu, Frankie Leung, Tak-Wing Lau, Hung-Fat Tse, Karen Siu-Ling Lam, Chung-Wah Siu

**Affiliations:** 1 Division of Endocrinology & Metabolism, Department of Medicine, Li Ka Shing Faculty of Medicine, The University of Hong Kong, Hong Kong SAR, Hong Kong; 2 Division of Cardiology, Department of Medicine, Li Ka Shing Faculty of Medicine, The University of Hong Kong, Hong Kong SAR; Hong Kong; 3 Cardiology Department, The Second People’s Hospital of Chengdu, Chengdu, China; 4 Division of Urology, Department of Surgery, Li Ka Shing Faculty of Medicine, The University of Hong Kong, Hong Kong SAR; Hong Kong; 5 Department of Orthopedics and Traumatology, Li Ka Shing Faculty of Medicine, The University of Hong Kong, Hong Kong SAR, Hong Kong; Turun Yliopisto, FINLAND

## Abstract

**Objective:**

Androgen deprivation therapy (ADT) increases fracture risk in men with carcinoma of the prostate, but little is known about the fracture risk for different types of ADT. We studied the fracture risk amongst Chinese patients with carcinoma of the prostate prescribed different ADT regimens.

**Subjects and methods:**

This was a single-centered observational study that involved 741 patients with carcinoma of the prostate from January 2001 to December 2011.

**Results:**

After a median follow-up of 5 years, 71.7% of the study cohort received ADT and the incidence rate of fracture was 8.1%. Multivariable Cox regression analysis revealed that use of ADT was significantly associated with risk of incident fracture (Hazard Ratio [HR] 3.60; 95% Confidence Interval [95% CI] 1.41–9.23; *p* = 0.008), together with aged >75 years and type 2 diabetes. Compared with no ADT, all three types of ADT were independently associated with the risk of incident fracture: anti-androgen monotherapy (HR 4.47; 95% CI 1.47–13.7; *p* = 0.009), bilateral orchiectomy ± anti-androgens (HR 4.01; 95% CI 1.46–11.1; *p* = 0.007) and luteinizing hormone-releasing hormone agonists ± anti-androgens (HR 3.16; 95% CI 1.18–8.43; *p* = 0.022). However, there was no significant difference in the relative risks among the three types of ADT.

**Conclusions:**

Fracture risk increases among all types of ADT. Clinicians should take into account the risk-benefit ratio when prescribing ADT, especially in elderly patients with type 2 diabetes.

## Introduction

Carcinoma of the prostate, a classic hormone-dependent malignancy, is the second most frequent cancer diagnosed and is the sixth most common cause of death in men worldwide.[1,2] In Hong Kong, carcinoma of the prostate ranks as the third leading cancer in men, accounting for 6.5% of all cancers in those aged 45–64 years and 17% in those aged 65–74 years.[3] Over the last decade, the incidence of prostate carcinoma has been steadily increasing by 4.6% per annum, probably related to the aging population as well as a more westernized lifestyle in Hong Kong.[4]

Because it is androgen dependent, androgen deprivation therapy (ADT) is the mainstay systemic treatment in patients with metastatic carcinoma of the prostate.[5] It has also been widely used amongst those with less advanced disease.[6,7] To date, androgen deprivation can be achieved in several ways: surgically by performing bilateral orchiectomy, or pharmacologically with gonadotrophin-releasing hormone (GnRH) agonists such as luteinizing hormone-releasing hormone agonists (LHRHa), GnRH antagonists, and non-steroidal anti-androgen drugs such as bicalutamide and flutamide. Since many patients with carcinoma of the prostate often have a favorable prognosis and not uncommonly live a near-normal lifespan, the benefits of any treatment that aims to further improve long-term outcome should outweigh any potential treatment-associated adverse effects. The metabolic effects as well as the cardiovascular consequences of ADT have been increasingly recognized.[8] More recently, clinical data have suggested a higher fracture risk amongst patients with carcinoma of the prostate prescribed ADT.[9] This can lead to impaired quality of life [10] and/or even mortality.[11] Nonetheless previous studies that demonstrated ADT-associated fracture risks often focused on an individual type of ADT such as bilateral orchiectomy [12], or LHRH agonists [13], [14], or considered several types of ADT as a whole.[15] Given the different mechanisms of achieving androgen deprivation, it is conceivable that different types of ADT might confer a differential risk of fracture. The aim of this study was to evaluate the fracture risk for different forms of ADT [16] using data from a retrospective cohort of Chinese patients with carcinoma of the prostate.

## Methods

### Patients

Patients with a diagnosis of carcinoma of the prostate who were followed up at Queen Mary Hospital, Hong Kong between January 2001 and December 2011, were identified through the computer-based clinical management system. Patients were excluded if they had previously documented osteoporosis and/or fracture(s) at the time of diagnosis of prostate carcinoma. Patients were then classified according to the type of ADT: (1) No ADT, (2) Bilateral orchiectomy with or without anti-androgens, (3) LHRHa with or without anti-androgens, or (4) Anti-androgen monotherapy such as cyproterone, flutamide, bicalutamide and ketoconazole. Those who were prescribed LHRHa or anti-androgen monotherapy had to have received at least 6 months of treatment to be included in this study. The final analysis included 741 patients with carcinoma of the prostate.

### Design

This was a single-centered, observational study conducted at a university hospital in Hong Kong. The study was approved by the Institutional Review Board of the University of Hong Kong / Hospital Authority Hong Kong West Cluster. Given the registry nature of the study, informed consent was not obtained from patients; nonetheless all patient records and information were rendered anonymous prior to analysis. Clinical data pertaining to fracture risk including demographics (age and smoking), medical history (co-morbidities including diabetes mellitus, hypertension, hypercholesterolemia, coronary artery disease, and stroke) and drug history (use of anti-osteoporotic medications, calcium or vitamin D supplements) were recorded at baseline.

### Outcomes, variables and data source

The primary outcome was hospital admission for fracture. An incident fracture was defined as the first recorded diagnosis of fracture verified from the Hospital Authority database as of 31^st^ December 2014. Diagnoses of fracture were based on International Classification of Diseases ninth version (ICD-9) (733, 805–809, 810–819 and 820–829). All medical records were reviewed and the endpoints were adjudicated such that traumatic or pathological fractures, which were based on bone scan or histology, were excluded. Data were retrieved from the medical records and discharge summaries of the territory-wide information network of all public hospitals in Hong Kong.

### Statistical analyses

Data that were not normally distributed, as determined using Kolmogorov-Smirnov test, were natural-logarithmically transformed to obtain near normality before analysis. Values are reported as mean ± standard deviation (SD) or median with inter-quartile range (IQR) as appropriate. Chi-square test and ANOVA were used for comparison of categorical and continuous variables, respectively. Multivariable Cox regression analysis was used to estimate the hazard ratio (HR) and 95% confidence interval (CI) for incident fractures. The variables included in Cox regression models were those that were statistically significant in univariate analysis. In all statistical tests, two-sided p-value <0.05 was considered significant. All analyses were performed with IBM SPSS Statistics 19.

## Results

A total of 741 subjects with carcinoma of the prostate were recruited. The mean age was 72.9±8.5 years. Table 1 summarizes their baseline clinical characteristics: 375 patients (50.6%) had hypertension; 125 patients (16.9%) had diabetes mellitus; 73 patients (9.9%) had coronary artery disease; and 41 (5.5%) had stroke. Amongst these, 210 patients (28.3%) had not received any ADT, and the remaining 530 patients (71.7%) were prescribed various types of ADT. Bilateral orchiectomy had been performed in 225 patients (30.4%) with or without non-steroidal anti-androgen drugs; 245 patients (33.1%) had LHRHa with or without non-steroidal anti-androgen drugs; and 61 (8.2%) were prescribed non-steroidal anti-androgen drugs only.

**Table 1:** Baseline characteristics of patients.

Baseline variables		Incident fracture		
	All (N = 741)	Yes (N = 60)	No (N = 681)	p-value
Age (years)	72.9±8.5	76.8±8.1	72.6±8.5	**<0.001**
Current smoker, n (%)	292 (39.4)	30 (50.0)	262 (38.5)	0.080
Diabetes mellitus, n (%)	125 (16.9)	15 (25.0)	110 (16.2)	0.079
Hypertension, n (%)	375 (50.6)	32 (53.3)	343 (50.4)	0.660
Hypercholesterolemia, n (%)	126 (17.0)	5 (8.3)	121 (17.8)	0.062
Coronary artery disease, n (%)	73 (9.9)	6 (10.0)	67 (9.8)	0.968
Stroke, n (%)	41 (5.5)	6 (10.0)	35 (5.1)	0.114
Use of steroid, n (%)	34 (4.6)	2 (3.3)	32 (4.7)	0.631
Use of calcium or vitamin D supplements, n (%)	116 (15.7)	8 (13.3)	108 (15.9)	0.713
Use of anti-osteoporotic medications, n (%)	56 (7.6)	1 (1.7)	55 (8.1)	0.076
Types of ADT				**0.002**
None, n (%)	210 (28.3)	5 (8.3)	205 (30.1)	
Orchiectomy ± anti-androgens, n (%)	225 (30.4)	22 (36.7)	203 (29.8)	
LHRHa ± anti-androgens, n (%)	245 (33.1)	24 (40.0)	221 (32.5)	
Anti-androgens alone, n (%)	61 (8.2)	9 (15.0)	52 (7.6)	

ADT, Androgen deprivation therapy; LHRHa, Luteinizing hormone releasing hormone agonists.

**Fig 1:**
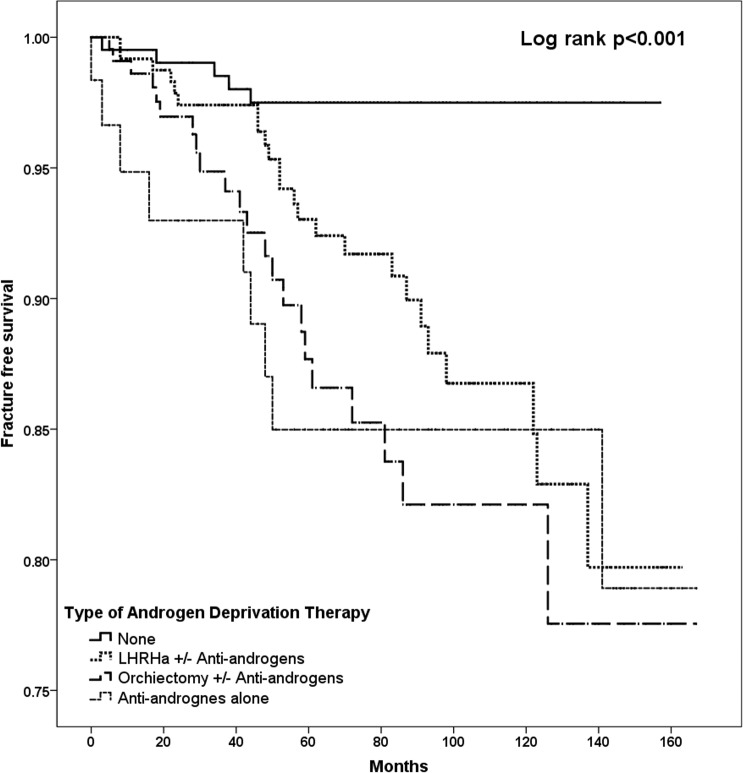
Kaplan Meier analysis of fracture-free survival among patients prescribed various types of ADT.

After a median follow-up of 5 years, 60 patients had an incident fracture (8.1%). Patients with incident fracture were older compared with patients without incident fracture (76.8±8.1 years, vs. 72.6±8.5 years, *p*<0.001), but there were no other statistically significant differences in terms of their smoking status, co-morbid medical conditions, or use of steroids during the follow-up period. Nonetheless patients with incident fracture were more likely to be prescribed ADT compared with those without fracture (91.7% *vs*. 69.9%, *p* = 0.002). It is noteworthy that of these 60 incident fractures, 55 occurred in patients prescribed ADT (10.3%), and only 5 occurred in patients not on ADT (2.4%). The annual incidence of fracture was 19.8 per 10,000 persons-years amongst patients on ADT compared with only 3.98 per 10,000 persons-years amongst those not on ADT. On multivariable Cox regression analysis, only age older than 75 years (HR: 2.93; 95% CI: 1.01–8.44; *p* = 0.046), presence of diabetes mellitus (HR: 1.83; 95% CI: 1.01–3.37; *p* = 0.045) and use of ADT (HR 3.60; 95% CI: 1.41–9.23; *p* = 0.008) were associated with increased risk of incident fracture. (Table 2) In addition, compared with no ADT, all three types of ADT were independently associated with an increased risk of incident fracture: anti-androgen monotherapy (HR: 4.47; 95% CI: 1.47–13.7; *p* = 0.009), bilateral orchiectomy ± anti-androgens (HR: 4.01; 95% CI: 1.46–11.1; *p* = 0.007), and LHRHa ± anti-androgens (HR: 3.16; 95% CI: 1.18–8.43; *p* = 0.022). However, there was no significant difference in the relative risks among the three types of ADT (Bilateral orchiectomy ± anti-androgens vs. LHRHa ± anti-androgens, p = 0.994; bilateral orchiectomy ± anti-androgens vs. anti-androgens alone, p = 0.202; LHRHa ± anti-androgens vs. anti-androgens monotherapy, p = 0.200). Fig 1 depicts the Kaplan Meier analysis of incident fracture amongst patients with carcinoma of the prostate prescribed different ADT regimens (log rank: 17.327, *p*<0.001). The results were similar after excluding those patients prescribed LHRHa alone and those who had bilateral orchiectomy without anti-androgens. Amongst those with bilateral orchiectomy or LHRHa, the addition of anti-androgen monotherapy did not increase the risk of incident fracture (bilateral orchiectomy HR: 1.30; 95% CI: 0.72–2.33; *p* = 0.383; and LHRHa HR: 1.36; 95% CI: 0.32–5.76; *p* = 0.676).

**Table 2:** Association of baseline factors with incident fracture.

Baseline variables	Number of fractures	Univariate analysis		Multivariable Stepwise Cox regression	
		HR (95% CI)	*p*-value	HR (95% CI)	*p*-value
Age at diagnosis			0.001**		0.038*
<65	4	Reference		Reference	
65–75	20	2.04 (0.70–5.97)	0.194	1.63 (0.55–4.82)	0.374
>75	36	4.45 (1.58–12.5)	0.005**	2.93 (1.01–8.44)	0.046*
Current smoker	30	1.73 (1.05–2.88)	0.033*		
Diabetes mellitus	15	1.95 (1.09–3.51)	0.025*	1.83 (1.01–3.37)	0.045*
Hypertension	32	1.20 (0.72–1.99)	0.485		
Hypercholesterolemia	5	0.45 (0.18–1.13)	0.090		
Coronary artery disease	6	1.11 (0.48–2.57)	0.815		
Stroke	6	2.37 (1.02–5.50)	0.045*		
Use of steroid	2	0.84 (0.20–3.43)	0.805		
Use of calcium or vitamin D supplements	8	0.86 (0.41–1.80)	0.683		
Use of anti-osteoporotic medications	1	0.33 (0.05–2.37)	0.269		
Use of ADT	55	5.01 (2.00–12.5)	0.001**		
Type of ADT			0.003**		0.041*
None	5	Reference		Reference	
Orchiectomy ± anti-androgens	22	6.09 (2.30–16.1)	<0.001***	4.01 (1.46–11.1)	0.007**
LHRHa ± anti-androgens	24	4.13 (1.57–10.8)	0.004**	3.16 (1.18–8.43)	0.022*
Anti-androgens alone	9	5.79 (1.93–17.4)	0.002**	4.47 (1.47–13.7)	0.009**

*p<0.05

**p<0.01

***p<0.001; Bilateral orchiectomy ± anti-androgens vs. LHRHa ± anti-androgens, p = 0.994; bilateral orchiectomy ± anti-androgens vs. anti-androgens alone, p = 0.202; LHRHa ± anti-androgens vs. anti-androgens monotherapy, p = 0.200); HR (95% CI), hazard ratios; ADT, Androgen deprivation therapy; LHRHa, Luteinizing hormone releasing hormone agonists. Multivariable Cox regression included all variables with p <0.05 in univariate analysis.

## Discussion

We compared the incidence rate of fracture in a contemporary cohort of patients with carcinoma of the prostate in regard to their ADT regimen. Our results identified several risk factors for incident fracture in this cohort of Chinese patients with carcinoma of the prostate, including aged more than 75 years, type 2 diabetes, and the use of ADT. Furthermore, all three forms of most commonly used ADT regimens were associated with significant increased risk of incident fracture, although there was no significant difference in the relative risks among them.

As is well known, patients with osteoporotic fractures suffer from significant morbidity and mortality. Up to 20% of patients with hip or vertebral fractures die within 5 years of the event, and the mortality rate is highest in men aged over 75 years with multiple co-morbid conditions. Moreover, among those who suffer hip fracture, 40% are reportedly unable to walk independently one year after the event, 60% require assistance in at least one essential activity of daily living, and 80% have difficulty with at least one instrumental activity of daily living.[17] Since about 75% of prostate cancers occur in elderly men aged 65 years or older, most are already at increased risk of osteoporotic fractures on the basis of age alone.[18] The use of ADT further accelerates this age-related bone loss with resultant clinical fractures.[18]

Although the incidence of fracture worldwide increases progressively with age, the incidence rate nonetheless varies across geographic regions and different ethnicities. In a report on the incidence of hip fractures involving 33 countries or regions, the highest annual age-sex-standardized hip fracture rates were found in Scandinavian countries (up to 47.74 per 10,000 population), compared with most European countries (up to 30.62 per 10,000 population) and the US (up to 26.47 per 10,000 population). In Asia, the rate was higher in Hong Kong (23.58 per 10,000 population), Taiwan (26.99 per 10,000 population) and Singapore (24.21 per 10,000 population), than in Iran (9.58 per 10,000 population) and Mainland China (6.70–13.16 per 10,000 population). Furthermore, among men in the US, white men had a higher hip fracture rate than Asians and blacks. [19] Differences in peak bone mass and skeletal geometry, genetic, lifestyle and environmental factors are all possible explanations for these geographical variations.[19] [20] These racial differences were also evident among men receiving ADT. In a study comparing the bone mineral density (BMD) and fracture incidence between African American and Caucasian men prescribed ADT, Caucasian men tended to develop more fractures during ADT. Since the decline in BMD was similar between the two groups, this might be attributed to the higher baseline BMD in African Americans.[21] Nonetheless although literature is scarce, Caucasian men receiving ADT tended to have a higher incidence of osteoporosis than Asians. In a study involving 58 Japanese men who received ADT, the incidence of osteoporosis by BMD was 10.8%, lower than that reported in a Caucasian series.[22] In our study, the incident fracture rate was 10.3% among men who received ADT, similar to the 9.8% reported in a recent Taiwan study of 17359 men with carcinoma of the prostate.[23] This again raises the possibility that geographical variations and racial differences might also play a role in determining susceptibility to ADT-related bone loss and fracture incidence in men prescribed ADT for carcinoma of the prostate.

There are at least four different types of ADT, each with its own strengths and limitations.[16] Nonetheless, to date, there is a paucity of data directly comparing their associated fracture risk. Bilateral orchiectomy and LHRHa are two common forms of ADT offered to patients with metastatic carcinoma of the prostate or locally advanced disease. Either of them alone has been shown to increase fracture risk in previous studies [12,13], but few have compared their relative risks.[23] In a study involving more than 50,000 men with carcinoma of the prostate, among those prescribed ADT, 19% suffered from a fracture. The relative risk was 1.54 (95% CI 1.42–1.68) for orchiectomy and 1.45 (95% CI 1.36–1.56) for those who received more than nine doses of LHRHa. Nevertheless this study also included possible pathological fractures resulting from bone metastases.[9] It was recently shown in a Chinese cohort of 17,359 patients with carcinoma of the prostate in Taiwan that the rate of fracture-free survival was highest in those without ADT, followed by those prescribed LHRHa, and lowest among those who had bilateral orchiectomy ± LHRHa. Nonetheless, there was still no exact comparison of fracture risk between these two common forms of ADT.[23]

**Fig 2:**
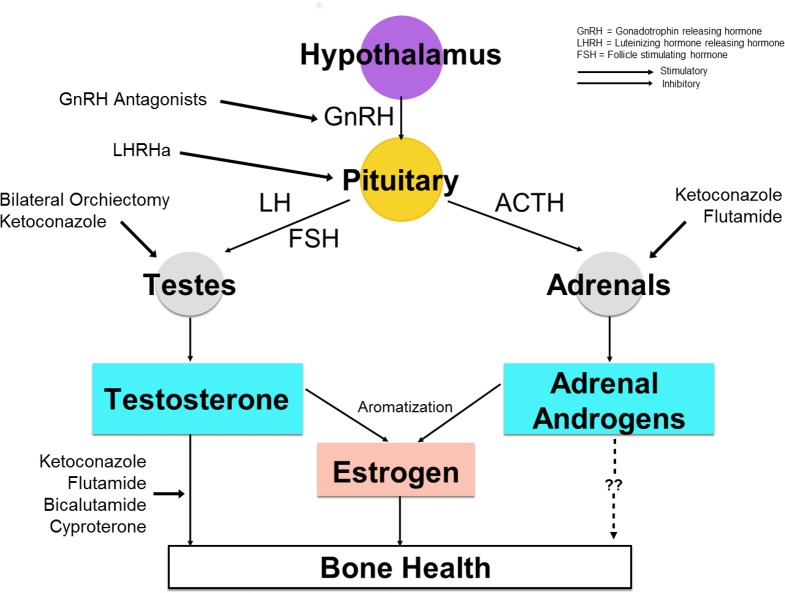
Association of different types of androgen deprivation therapy with bone health.

In our cohort, all forms of ADT, including bilateral orchiectomy ± anti-androgens, LHRHa ± anti-androgens, and anti-androgens monotherapy were associated with a significant increase in incident fracture. However, there was no significant difference in the relative risks among the three modalities. In men, testosterone is the most important androgen and is closely related to bone health, either directly affecting osteogenesis [24] or indirectly through aromatization to estradiol.[25] To date, androgen deprivation can be achieved in several ways, acting at different levels of the hypothalamic-pituitary-gonadal axis: Bilateral orchiectomy brings about a rapid, irreversible and pronounced suppression of serum testosterone levels, LHRHa causes suppression of LH, FSH and serum testosterone over time, and most anti-androgens compete with serum androgens at the receptor level. Fig 2 depicts the association of different types of ADT with bone health. Our results highlighted that androgen deprivation, irrespective of the mechanisms through which it is achieved, produced deleterious effects on bone health. In a prospective study involving 618 men with carcinoma of the prostate, the use of bicalutamide, a potent non-steroidal anti-androgen, was shown to maintain BMD while LHRHa decreased BMD. The authors suggested that this was likely an effect of increased estradiol levels produced from aromatization of the increased amount of serum testosterone available under androgen receptor blockade.[26] In our cohort, only 11% of subjects were prescribed bicalutamide and a large majority of subjects were on flutamide as anti-androgen monotherapy. In contrast to bicalutamide with no effect on adrenal androgen, flutamide was shown to significantly reduce the level of dehydroepiandrosterone (DHEA) in addition to androgen receptor blockade.[27] However, whether reduction in adrenal androgen contributes to fracture risk in patients with carcinoma of the prostate remains to be confirmed by further studies. (Fig 2)

In our study, diabetes mellitus was also associated with increased risk of fracture in patients with carcinoma of the prostate. Type 2 diabetes mellitus has been linked with bone fragility via multiple proposed mechanisms, including reduced insulin growth factor-1 (IGF1) production, vitamin D deficiency and increased circulating advanced glycation end-products (AGE). In addition, insulin resistance is associated with low testosterone levels that may further aggravate bone fragility in men receiving ADT.[28]

Our findings certainly have important implications in the management of carcinoma of the prostate. Several decades have passed since the discovery of ADT. Despite the increasingly recognized long term side effects of ADT, be it cardiovascular, metabolic or skeletal complications, there remains an important role of ADT in patients suffering from metastatic carcinoma of the prostate and those with high-risk and locally advanced disease, in terms of both symptom palliation and survival benefits.[29,30] Our study provides additional information about the bone-related side effects of ADT, highlighting that although a form of ADT might be chosen for their merits of reversibility (e.g. LHRHa), or rapid and pronounced testosterone suppression (e.g. bilateral orchiectomy), or compliance (e.g. anti-androgen monotherapy), their deleterious effects on bone health occurred at a similar extent, hence more comprehensive counseling about the anticipated risks and related preventive measures could be afforded to patients who are prescribed ADT. On the other hand, for patients with less advanced disease, understanding more about these fracture risks enables clinicians to derive a clearer risk-benefit ratio while prescribing ADT for their patients, especially in the elderly population and those with type 2 diabetes, who are already at a higher risk of incident fracture. This is particularly important since patient’s quality of life can be significantly affected by the occurrence of an ADT-related clinical fracture or cardiovascular disease.

### Limitations

The study was limited by its single-centered observation design and the relatively small sample size. Due to registry design, the selection of ADT was not in a randomized controlled fashion. Patients who were prescribed ADT were in some way different to their counterparts who were not treated with ADT as judged by their attending urologists. Thus selection bias was possible. Importantly, given the nature of the study, we lacked the ability and access to have all other factors described, quantified and compared, including some important data such as bone mineral density, alcohol consumption, socio-economic status, serum testosterone level that may influence the treatment choice as well as the nominal fracture risk. In addition, some newer agents for ADT such as GnRH antagonists or abiraterone, were not available during the study period. While we carefully ascertained all fracture events by detailed examination of hospital records, laboratory and imaging results, we did not include patients with a milder form of fracture who were not hospitalized.

Nonetheless, our study has compared the fracture risks of various types of ADT among Chinese patients with carcinoma of the prostate. As all forms of ADT increase incident fracture without demonstrable difference in relative risks among them, we highlight the need for preventive measures to optimize bone health before and during ADT, irrespective of the types of ADT prescribed. These include adequate calcium and vitamin D intake and/or supplements, as well as regular weight-bearing exercises. Current clinical practice guidelines recommend BMD evaluation with dual energy X-ray absorptiometry in men aged 50 or above who were hypogonadal or prescribed LHRHa.[31] Moreover, in men receiving ADT, measuring radius BMD might be particularly helpful as BMD of the ultra distal radius was found to decline at a greater extent than the spine and hip.[31,32] In those who are at high risk of fracture, pharmacological agents like bisphosphonate therapy, either oral or intravenous, or denosumab can be considered [31], so that ADT will improve the overall survival of patients with prostate cancer without compromising their quality of life.

## Supporting information

S1 Dataset Data as supporting information file. (XLSX)

## References

[1] Torre LA, Bray F, Siegel RL, Ferlay J, Lortet-Tieulent J, et al (2015) Global cancer statistics, 2012. CA Cancer J Clin 65: 87–108. 10.3322/caac.21262 25651787

[2] Jemal A, Bray F, Center MM, Ferlay J, Ward E, et al (2011) Global cancer statistics. CA Cancer J Clin 61: 69–90. 10.3322/caac.20107 21296855

[3] Ngan. RK (2014) Overview of 2012 Hong Kong Cancer Statistic. Hong Kong Cancer Registry, Hospital Authority.

[4] Xie WC, Chan MH, Mak KC, Chan WT, He M (2012) Trends in the incidence of 15 common cancers in Hong Kong, 1983–2008. Asian Pac J Cancer Prev 13: 3911–3916. 23098492 10.7314/apjcp.2012.13.8.3911

[5] Hussain S, Gunnell D, Donovan J, McPhail S, Hamdy F, et al (2008) Secular trends in prostate cancer mortality, incidence and treatment: England and Wales, 1975–2004. BJU Int 101: 547–555. 10.1111/j.1464-410X.2007.07338.x 18190630 PMC2765909

[6] Bolla M, Van Tienhoven G, Warde P, Dubois JB, Mirimanoff RO, et al (2010) External irradiation with or without long-term androgen suppression for prostate cancer with high metastatic risk: 10-year results of an EORTC randomised study. Lancet Oncol 11: 1066–1073. 10.1016/S1470-2045(10)70223-0 20933466

[7] Payne H, Mason M (2011) Androgen deprivation therapy as adjuvant/neoadjuvant to radiotherapy for high-risk localised and locally advanced prostate cancer: recent developments. Br J Cancer 105: 1628–1634. 10.1038/bjc.2011.385 22009028 PMC3242586

[8] Huang G, Yeung CY, Lee KK, Liu J, Ho KL, et al (2014) Androgen deprivation therapy and cardiovascular risk in chinese patients with nonmetastatic carcinoma of prostate. J Oncol 2014: 529468 10.1155/2014/529468 24803931 PMC3997904

[9] Shahinian VB, Kuo YF, Freeman JL, Goodwin JS (2005) Risk of fracture after androgen deprivation for prostate cancer. N Engl J Med 352: 154–164. 10.1056/NEJMoa041943 15647578

[10] Saad F, Olsson C, Schulman CC (2004) Skeletal morbidity in men with prostate cancer: quality-of-life considerations throughout the continuum of care. Eur Urol 46: 731–739; discussion 739–740. 10.1016/j.eururo.2004.08.016 15548440

[11] Oefelein MG, Ricchiuti V, Conrad W, Resnick MI (2002) Skeletal fractures negatively correlate with overall survival in men with prostate cancer. J Urol 168: 1005–1007. 10.1097/01.ju.0000024395.86788.cc 12187209

[12] Melton LJ 3rd, Alothman KI, Khosla S, Achenbach SJ, Oberg AL, et al (2003) Fracture risk following bilateral orchiectomy. J Urol 169: 1747–1750. 10.1097/01.ju.0000059281.67667.97 12686824

[13] Smith MR, Lee WC, Brandman J, Wang Q, Botteman M, et al (2005) Gonadotropin-releasing hormone agonists and fracture risk: a claims-based cohort study of men with nonmetastatic prostate cancer. J Clin Oncol 23: 7897–7903. 10.1200/JCO.2004.00.6908 16258089

[14] Lopez AM, Pena MA, Hernandez R, Val F, Martin B, et al (2005) Fracture risk in patients with prostate cancer on androgen deprivation therapy. Osteoporos Int 16: 707–711. 10.1007/s00198-004-1799-7 15714259

[15] Teoh JY, Chiu PK, Chan SY, Poon DM, Cheung HY, et al (2015) Androgen deprivation therapy, diabetes and poor physical performance status increase fracture risk in Chinese men treated for prostate cancer. Aging Male: 1–6. 26004988 10.3109/13685538.2015.1046043

[16] Schroder F, Crawford ED, Axcrona K, Payne H, Keane TE (2012) Androgen deprivation therapy: past, present and future. BJU Int 109 Suppl 6: 1–12. 10.1111/j.1464-410X.2012.11215.x 22672120

[17] Cooper C (1997) The crippling consequences of fractures and their impact on quality of life. Am J Med 103: 12S–17S; discussion 17S-19S. 10.1016/s0002-9343(97)90022-x 9302893

[18] Grossmann M, Zajac JD (2011) Androgen deprivation therapy in men with prostate cancer: how should the side effects be monitored and treated? Clin Endocrinol (Oxf) 74: 289–293. 21092052 10.1111/j.1365-2265.2010.03939.x

[19] Cheng SY, Levy AR, Lefaivre KA, Guy P, Kuramoto L, et al (2011) Geographic trends in incidence of hip fractures: a comprehensive literature review. Osteoporos Int 22: 2575–2586. 10.1007/s00198-011-1596-z 21484361

[20] Cummings SR, Cauley JA, Palermo L, Ross PD, Wasnich RD, et al (1994) Racial differences in hip axis lengths might explain racial differences in rates of hip fracture. Study of Osteoporotic Fractures Research Group. Osteoporos Int 4: 226–229. 7949753 10.1007/BF01623243

[21] Morgans AK, Hancock ML, Barnette KG, Steiner MS, Morton RA, et al (2012) Racial differences in bone mineral density and fractures in men receiving androgen deprivation therapy for prostate cancer. J Urol 187: 889–893. 10.1016/j.juro.2011.10.136 22245322 PMC3671868

[22] Wang W, Yuasa T, Tsuchiya N, Maita S, Kumazawa T, et al (2008) Bone mineral density in Japanese prostate cancer patients under androgen-deprivation therapy. Endocr Relat Cancer 15: 943–952. 10.1677/ERC-08-0116 18667685

[23] Wu CT, Yang YH, Chen PC, Chen MF, Chen WC (2015) Androgen deprivation increases the risk of fracture in prostate cancer patients: a population-based study in Chinese patients. Osteoporos Int. 10.1007/s00198-015-3135-9 25990353

[24] Garcia-Fontana B, Morales-Santana S, Varsavsky M, Garcia-Martin A, Garcia-Salcedo JA, et al (2014) Sclerostin serum levels in prostate cancer patients and their relationship with sex steroids. Osteoporos Int 25: 645–651. 10.1007/s00198-013-2462-y 23903956

[25] Cauley JA, Ewing SK, Taylor BC, Fink HA, Ensrud KE, et al (2010) Sex steroid hormones in older men: longitudinal associations with 4.5-year change in hip bone mineral density—the osteoporotic fractures in men study. J Clin Endocrinol Metab 95: 4314–4323. 10.1210/jc.2009-2635 20554716 PMC2936055

[26] Wadhwa VK, Weston R, Mistry R, Parr NJ (2009) Long-term changes in bone mineral density and predicted fracture risk in patients receiving androgen-deprivation therapy for prostate cancer, with stratification of treatment based on presenting values. BJU Int 104: 800–805. 10.1111/j.1464-410X.2009.08483.x 19338564

[27] Nakai Y, Tanaka N, Anai S, Miyake M, Tatsumi Y, et al (2015) A Randomized Control Trial Comparing the Efficacy of Antiandrogen Monotherapy: Flutamide vs. Bicalutamide. Horm Cancer. 10.1007/s12672-015-0226-1 PMC10355925 26024831

[28] Moseley KF (2012) Type 2 diabetes and bone fractures. Curr Opin Endocrinol Diabetes Obes 19: 128–135. 10.1097/MED.0b013e328350a6e1 22262002 PMC4753802

[29] Mottet N, Bellmunt J, Bolla M, Briers E, Cumberbatch MG, et al (2016) EAU-ESTRO-SIOG Guidelines on Prostate Cancer. Part 1: Screening, Diagnosis, and Local Treatment with Curative Intent. Eur Urol. 10.1016/j.eururo.2016.08.003 27568654

[30] Pagliarulo V, Bracarda S, Eisenberger MA, Mottet N, Schroder FH, et al (2012) Contemporary role of androgen deprivation therapy for prostate cancer. Eur Urol 61: 11–25. 10.1016/j.eururo.2011.08.026 21871711 PMC3483081

[31] Watts NB, Adler RA, Bilezikian JP, Drake MT, Eastell R, et al (2012) Osteoporosis in men: an Endocrine Society clinical practice guideline. J Clin Endocrinol Metab 97: 1802–1822. 10.1210/jc.2011-3045 22675062

[32] Mittan D, Lee S, Miller E, Perez RC, Basler JW, et al (2002) Bone loss following hypogonadism in men with prostate cancer treated with GnRH analogs. J Clin Endocrinol Metab 87: 3656–3661. 10.1210/jcem.87.8.8782 12161491

